# Beyond the negative view of music teacher stress: how stress enhances occupational well-being through teacher agency

**DOI:** 10.3389/fpsyg.2026.1865707

**Published:** 2026-09-02

**Authors:** Zhuowei Li, Xiaobo Han, Wei Wei, Xiaoyu Zhang

**Affiliations:** 1 School of Music, Yeungnam University, Gyeongsan, Republic of Korea; 2 Shanxi Jinzhong Institute of Technology, Jinzhong, China; 3 School of Textile Garment and Design, Suzhou Institute of Technology, Changshu, China; 4 USCI Graduate Business School, UCSI University, Cheras, Malaysia

**Keywords:** occupational well-being, serial mediation, sustainability, teacher agency, teacher stress

## Abstract

This study aims to examine the relationship between teacher stress and occupational well-being among Chinese in-service primary and secondary school music teachers, with particular attention to its non-linear effects and the serial mediating roles of teacher agency and job burnout. A quantitative research design was adopted, and data were collected from 205 in-service primary and secondary school music teachers in China. Structural equation modeling and serial mediation analysis were employed to test the proposed hypotheses. The results indicate that music teacher stress exerts a positive direct association on occupational well-being and exhibits a U-shaped relationship. In addition, teacher stress does not have a significant direct association on job burnout but influences occupational well-being through teacher agency and a serial mediation pathway involving teacher agency and job burnout. By incorporating a non-linear perspective and a serial mediation framework, this study explains how music teacher stress influences occupational well-being through a mechanism centered on teacher agency and job burnout. Practically, the findings suggest that maintaining an appropriate level of stress and fostering music teacher agency are important for supporting teachers’ occupational well-being and sustainable professional development.

## 1 Introduction

### 1.1 Research background

Teacher occupational well-being has increasingly become an important topic in educational research, particularly in relation to the sustainable development of the education system. Its level is not only related to teachers’ individual development, but also directly affects teaching quality and students’ learning outcomes (Ortan et al., 2021; Pagán-Castaño et al., 2021). The Organisation for Economic Co-operation and Development (OECD, 2020) has explicitly identified teacher well-being as a key component in sustaining educational quality over the long term (Viac and Fraser, 2020). However, despite its growing importance, teachers’ occupational well-being is facing serious challenges globally. Specifically, teachers are employed within a system characterized by contract-based hiring and performance-driven promotion (Knights and Clarke, 2014; Si, 2024). This system intensifies competition and work pressure, thereby undermining the sustainable maintenance of occupational well-being (Xu, 2021; Cheng et al., 2024). This problem is particularly pronounced among music teachers, whose workload and stress levels are often higher due to their additional responsibilities such as rehearsal and performance organization (Powell, 2021; Ding and Yan, 2024). Therefore, teacher stress has become an important factor influencing the sustainability of occupational well-being.

### 1.2 Problem statement and research gap

Existing research has predominantly examined the relationship between music teacher stress and occupational well-being through the lens of the Job Demands–Resources (JD-R) model. Within this framework, prior studies have largely focused on the resource depletion pathway, suggesting that teacher stress arises when perceived job demands exceed individuals’ coping capacity (Kyriacou, 2001; Collie, 2021). Role overload (Sandene, 1995), underutilization of professional skills (Scheib, 2003), and teaching responsibilities extending beyond teachers’ primary areas of expertise (National String Project Consortium, 2010) have all been identified as important sources of stress among music teachers. Such stress is assumed to continuously consume emotional and psychological resources, leading to emotional exhaustion, reduced work engagement, and ultimately lower levels of occupational well-being (Shao et al., 2025; Maslach and Leiter, 2000; Skaalvik and Skaalvik, 2018). This perspective primarily conceptualizes stress as a detrimental factor, emphasizing its erosive impact on individual resources. However, stress is not inherently negative. Drawing on the challenge–hindrance stress framework, LePine et al. (2004) argue that different types of stressors may generate distinct effects, with challenge stressors potentially stimulating intrinsic motivation and enhancing work engagement. These inconsistent findings suggest that the effect of music teacher stress on occupational well-being is not unidirectional but may operate through multiple pathways and even exhibit nonlinear patterns. This complexity may be particularly pronounced among music teachers, as their work often involves clearly defined goals and observable outcomes, such as rehearsals and performances (Powell, 2021), which give their professional responsibilities multiple dimensions (Taft, 2023). In addition, many music educators are required to undertake teaching responsibilities beyond their primary areas of specialization (Gillespie and Hamann, 1998; Groulx, 2016). Such responsibilities are more likely to be appraised by teachers as demanding and challenging job demands (Taft, 2023). Despite this, existing research has largely remained at the level of identifying variable relationships, generally assuming a linear relationship between teacher stress and occupational well-being, while lacking a systematic explanation of why and how teacher stress may produce differentiated or nonlinear effects. However, according to the Yerkes–Dodson law (Yerkes and Dodson, 1908), the effects of stress are not necessarily linear. Moderate levels of stress or arousal may facilitate individuals’ motivation and adaptive functioning, whereas excessively low or high levels of stress are more likely to impair performance and psychological functioning. This theoretical perspective suggests that the effects of teacher stress may vary depending on the level of stress. Therefore, the present study considers it necessary to further examine whether a potential nonlinear relationship exists between teacher stress and occupational well-being.

### 1.3 Theoretical foundation

To explain these complex mechanisms, the present study integrates the JD-R model and Self-Determination Theory (SDT) as its theoretical framework. The present study argues that the JD-R model and SDT play complementary roles in explaining how teacher stress influences occupational well-being, jointly describing a continuous psychological process through which teachers respond to work stress. Specifically, SDT mainly explains how teachers make adaptive responses to work stress through proactive regulation, whereas the JD-R model further explains how persistent job demands lead to job burnout through resource depletion. More specifically, teachers’ responses to work stress constitute a dynamic process. In this process, teachers initially regulate work stress through proactive regulation. However, when job demands persist and available resources become insufficient, such proactive regulation gradually becomes difficult to sustain and eventually develops into job burnout. Therefore, the present study does not conceptualize teacher agency and job burnout as two independent mediators; rather, they are regarded as two sequential psychological stages in teachers’ responses to work stress, based on which a serial mediation model is proposed. Integrating these two theoretical perspectives contributes to a more comprehensive understanding of the process of motivational regulation and the subsequent process of resource depletion under conditions of teacher stress.

According to SDT (Ryan and Deci, 2020), individuals are not passive recipients of external pressures but possess an inherent tendency toward growth, integration, and self-regulation. However, the expression of these proactive tendencies depends on the satisfaction of the basic psychological needs for autonomy, competence, and relatedness. From this perspective, individuals may actively respond to external pressures through self-regulatory behaviors rather than merely enduring them. Teacher agency, therefore, reflects teachers’ capacity to take purposeful action based on professional judgment, adapt their instructional practices, and shape their working environment within the constraints of their professional context (Biesta, 2015; Priestley et al., 2015). For example, music teachers may reorganize instructional activities or seek additional financial support to cope with occupational stress (Taft, 2023). Accordingly, teacher agency can be conceptualized as an adaptive and motivational pathway linking teacher stress and occupational well-being (Ryan and Deci, 2020; Tao and Gao, 2017).

Building on the integrated perspective of SDT and the JD-R mode, teacher agency is positioned prior to job burnout because it represents teachers’ early behavioral responses to stressful job demands. From the perspective of the JD-R model, employees’ work experiences are jointly determined by job demands and job resources (Bakker and Demerouti, 2017). Excessive job demands continuously consume teachers’ emotional and psychological resources, resulting in emotional exhaustion and job burnout, which subsequently undermine occupational well-being (Bakker and Demerouti, 2007). When stressful conditions persist and teachers’ proactive regulatory efforts are not adequately supported by sufficient job resources, accumulated resource depletion may gradually develop into a more stable state of job burnout. Consequently, job burnout is conceptualized in this study as the outcome of prolonged resource depletion occurring after teachers’ proactive regulatory efforts, as reflected in teacher agency.

Previous studies have typically focused on a single mediating mechanism, either examining job burnout as a mediator (Angelini et al., 2024) or emphasizing the buffering role of personal resources such as teacher self-efficacy (Han et al., 2020). To fill the research gaps mentioned above, this paper examines the linear and nonlinear effects of music teacher stress and incorporates teacher agency and burnout into a chain mediation model to systematically analyze the impact of music teachers’ work stress on occupational well-being and its underlying mechanisms. Theoretically, the present study integrates the JD-R model and SDT, explaining how teachers’ proactive regulatory process under stressful conditions may gradually give way to prolonged resource depletion when job demands persist, thereby providing a more coherent theoretical explanation for the serial mediating relationship between teacher agency and job burnout. Practically, the findings provide important implications for educational administrators to effectively regulate music teachers’ work stress, enhance teacher agency, and promote teachers’ sustainable career development.

## 2 Literature review and hypothesis development

### 2.1 Occupational well-being

Teacher well-being is generally considered a multidimensional construct encompassing individuals’ positive emotions and mental health (Van Horn et al., 2004), integrating physiological, social, emotional, and psychological aspects (Hascher and Waber, 2021). Existing research further indicates that teacher occupational well-being is not only reflected in individuals’ satisfaction and sense of accomplishment in the teaching context, but also in their subjective experience of realizing professional ideals and values during career development (Carine and Pablo, 2020; Tan, 2002; Kiltz et al., 2020). Simultaneously, teacher occupational well-being is closely related to positive emotions, work engagement, teacher resource support (Han and Gao, 2023), and a stable sense of professional achievement (Seligman, 2011; MacIntyre et al., 2019), and has a significantly associated with teachers’ physical and mental health, work performance, and educational quality (Zhen and Liu, 2023). However, in actual educational contexts, teacher occupational well-being is not stable but is easily affected by work-related factors, especially the continuous increase in work pressure, which poses a serious challenge.

Teachers often face heavy teaching loads, limited resource support, and insufficient professional development opportunities. These persistent stressful conditions are likely to be associated with emotional exhaustion and a reduced sense of accomplishment, thereby weakening their occupational well-being (Demir et al., 2025; Zhang et al., 2024). Furthermore, in an increasingly competitive educational environment, teachers may also face persistent psychological burdens and occupational anxiety, further increasing their perceived stress levels (Andrew, 2019). When teaching demands exceed individual capabilities, teachers are more likely to experience frustration and occupational stress, and is associated with a poorer professional experience (Levy-Vered and Nasser-Abu Alhija, 2015). From a more general perspective, these phenomena reflect the continuous depletion of individual resources by work stress; that is, high-intensity work demands are associated with the depletion of teachers’ emotional and psychological resources, thus negatively affecting their occupational well-being. This process is also widely supported in the JD-R model. However, this explanation based on resource depletion is still relatively simplistic and insufficient to fully reveal the complexity of the effects of teacher stress.

Existing research has explored the influencing factors of teachers’ occupational well-being from multiple perspectives, covering individual characteristics, psychological states, and organizational contexts. For example, teacher professional identity, mental health, and emotion regulation abilities are considered to contribute to improved occupational well-being (He et al., 2025; Benevene et al., 2019; Dreer, 2024), while external resources such as transformational leadership and school climate have been shown to improve teachers’ professional experience (Sun et al., 2025; Collie et al., 2012). Furthermore, individual traits and need satisfaction also influence occupational well-being to some extent (Zadok et al., 2024; Capone and Petrillo, 2020). However, these studies tend to focus on identifying the influencing factors themselves, paying less attention to the role of teacher stress within these factors. In contrast, some studies have begun to focus on the relationship between work stress and occupational well-being, generally suggesting that stress may be associated with lower occupational well-being through reduced work engagement and depletion emotional resources (Pakdee et al., 2025; Maslach and Leiter, 2000). Meanwhile, other studies have pointed out that different types of stress and individuals’ proactive behaviors may alter its effects to some extent (LePine et al., 2004; Nielsen and Munir, 2009). These studies indicate that the role of teacher stress in the formation of occupational well-being is neither singular nor linear, and its underlying mechanisms still lack systematic explanation (related studies are presented in Table 1).

**Table 1:** Factor loadings, CR, and AVE.

Constructs	Indicators	Loading	Composite reliability (CR)	Average variance extracted (AVE)
Teacher stress	TS1	0.765	0.908	0.553
	TS2	0.711		
	TS3	0.765		
	TS4	0.755		
	TS5	0.735		
	TS6	0.775		
	TS7	0.711		
	TS8	0.732		
Teacher agency	TA1	0.719	0.794	0.562
	TA2	0.786		
	TA3	0.742		
Job burnout	JB1	0.820	0.911	0.672
	JB2	0.829		
	JB3	0.824		
	JB4	0.828		
	JB5	0.797		
Occupational well-being	OWB1	0.810	0.900	0.599
	OWB2	0.783		
	OWB3	0.772		
	OWB4	0.770		
	OWB5	0.740		
	OWB6	0.768		

TS, Teacher stress; TA, Teacher agency; JB, Job burnout; OWB, Occupational well-being.

Source: authors own work.

Although existing research has explored teacher stress and its relationship with occupational well-being from multiple perspectives, a systematic explanation of the mechanisms through which stress operates remains lacking. Current research tends to focus on understanding the impact of stress from the perspective of resource consumption, paying less attention to the complex processes through which it may exert its effects via multiple pathways, and particularly neglecting individuals’ proactive regulatory behaviors in stressful situations. Therefore, it is necessary to further explore the internal mechanisms through which teacher stress affects occupational well-being from a more integrated theoretical perspective.

### 2.2 Teacher stress

Teacher stress, as a core variable in the work environment, has long been considered an important factor associated with job satisfaction. It is essentially a sustained psychological burden arising from intensified competition within the educational environment, reflecting the potential influence of the occupational context on teachers’ psychological states (Llorens and Salanova, 2014). Teacher stress has been classically defined as a response of negative emotional states such as anxiety, frustration, or depression arising from aspects of the teaching role (Kyriacou and Sutcliffe, 1978). In this study, it is conceptualized as perceived stress reflecting individuals’evaluation of the balance between risk and protective factors in their work environment, rather than objective workload alone. Starting from the JD-R model, increased job demands are understood to alter individuals’ resource input and psychological states at work, which in turn may shape their overall work experience and subjective feelings (Bakker and Demerouti, 2017). In the teaching context, high-intensity teaching tasks, performance pressure, and limited autonomy often constitute persistent sources of stress. These factors may be associated with reduced positive emotions and job satisfaction, thereby potentially undermining their occupational well-being. Therefore, existing research generally agrees that there is a significant relationship between teacher stress and occupational well-being (Shao et al., 2025). However, when individuals view stressful situations as manageable challenges, stress may stimulate their intrinsic motivation and work engagement, thereby improving their occupational experience to some extent (LePine et al., 2004). In this sense, teacher stress may both undermine individuals’ occupational experience and, under certain conditions, facilitate their positive development. More importantly, this unexpected finding also suggests a methodological possibility in that the “teacher stress’ measure used in this study may capture not only negative strain but also individuals’ subjective appraisal of work demands, including both stress-related experiences and, to some extent, challenge-related cognitions. Thus, having a comprehensive association on occupational well-being. Furthermore, according to the Yerkes–Dodson law (Yerkes and Dodson, 1908), different levels of stress may lead to different psychological and behavioral outcomes, suggesting that the relationship between teacher stress and occupational well-being may not be strictly linear. Building on prior research that predominantly emphasizes the detrimental role of stress, the present study continues to propose a negative linear relationship between teacher stress and occupational well-being as the primary hypothesis. At the same time, drawing on the challenge–hindrance stress framework and the above theoretical perspective, the present study further examines whether a potential nonlinear relationship exists between the two variables.

*H1*: Teacher Stress negatively associated with occupational well-being.

According to SDT, when individuals perceive stressful situations as controllable and challenging, their intrinsic motivation will be activated, thereby promoting more autonomous forms of behavior (Baard et al., 2004). In addition, existing research has shown that certain types of stress, such as challenge stressors, are positively correlated with motivation and work engagement (LePine et al., 2004). Although teacher stress was operationalized as a global construct in this study, existing research indicates that individuals’ responses to stressful situations are not consistent, but rather they integrate personal goals, values and beliefs with their evaluations of environmental demands and available resources, thereby forming different cognitive appraisals (Lazarus, 1991). Therefore, stress is not a single stimulus that directly determines behavioral outcomes, but rather is transformed into different psychological and behavioral response pathways through the cognitive appraisal process.

Specifically, when facing work-related stress, teachers may cognitively appraise certain job demands as opportunities to promote professional growth and believe that they are within their own capacity to cope. These job demands may be interpreted as work stressors with “challenge characteristics,” thereby stimulating more proactive behaviors (Cavanaugh et al., 2000). Under specific conditions, teacher stress may be positively associated with higher levels of teacher agency.

In contrast, when teachers appraise job demands as excessive, uncontrollable, or mismatched with their own resources, these stresses are more likely to be experienced as hindrance stressors, thereby leading to continuous depletion of psychological resources. From the perspective of the JD-R model, the combination of low decision latitude and high job demands is a core factor leading to psychological stress (Karasek, 1979), and is closely related to the development of job burnout (Maslach and Jackson, 1981). In the teaching context, insufficient teaching time, pressure to complete standardized curricula, and limited autonomy in instructional decisions (Oreck, 2004), as well as the additional time and effort required to prepare students for competitions (Powell, 2021), may continuously deplete teachers’ emotional and psychological resources. This long-term and cumulative high level of job demands is more likely to be appraised as hindrance stressors, thereby leading to emotional exhaustion and higher levels of job burnout (Bakker and Demerouti, 2017). When individuals form negative cognitive appraisals, teacher stress is more likely to be positively correlated with job burnout. Based on the above discussion, the following hypotheses are proposed:

*H2*: Teacher stress positively associated with teacher agency.

*H3*: Teacher stress positively related to job burnout.

### 2.3 Teacher agency

From the integrated perspective of SDT and the JD-R model, teachers are expected to initially respond to work-related stress through proactive self-regulation, whereas job burnout is more likely to emerge as a consequence of prolonged resource depletion. Therefore, the present study further examines the relationship between teacher agency and job burnout to establish a more comprehensive explanatory mechanism. Teacher agency is an important mechanism through which individuals actively regulate their work in response to job demands. In this study, teacher agency is conceptualized as teachers’ capacity to actively regulate their teaching practices and exercise professional autonomy within specific instructional contexts (Priestley et al., 2015). This definition primarily emphasizes teachers’ self-regulatory and autonomy-related capacities in managing instructional tasks, rather than their ability to directly change structural working conditions. From the perspective of the JD-R model, insufficient personal control under high job demands is associated with higher individuals’ vulnerability to psychological strain (Bakker and Demerouti, 2007). In the teaching context, when teachers lack the agency to cope with heavy teaching tasks and ongoing educational reforms, they are more likely to experience emotional exhaustion and job burnout (Skaalvik and Skaalvik, 2018). Therefore, higher levels of teacher agency are associated with a lower likelihood of experiencing job burnout.

From the perspective of SDT, teacher agency reflects individuals’ capacity to satisfy their basic psychological needs for autonomy and competence (Ryan and Deci, 2017). When teachers are able to actively regulate their teaching behaviors and effectively respond to work demands, they are more likely to experience a stronger sense of control and accomplishment, thereby enhancing occupational well-being (Day and Gu, 2014; Kim et al., 2022). In contrast, lower levels of teacher agency may weaken individuals’ positive work experience and undermine occupational well-being (Shao et al., 2025). Based on the above discussion, the following hypotheses are proposed:

*H4*: Teacher agency negatively related to job burnout.

*H5*: Teacher agency positively associated with occupational well-being.

### 2.4 Job burnout

According to SDT, when teachers perceive work-related stress, they initially engage in proactive self-regulation based on their professional judgment and autonomous motivation in order to adapt to job demands and maintain effective functioning. However, when stressful job demands persist and continuously exceed teachers’ available resources, these proactive efforts may gradually become insufficient, and prolonged resource depletion may eventually develop into job burnout. Accordingly, job burnout can be regarded as the psychological consequence of prolonged resource depletion in the stress process. Job burnout can be understood as a psychological state characterized by emotional exhaustion, depersonalization, and a reduced sense of personal accomplishment under prolonged work-related stress (Maslach and Jackson, 1981). Previous research has identified emotional exhaustion as the core dimension of job burnout. It is widely considered the earliest manifestation of burnout and may subsequently contribute to more severe depersonalization and diminished personal accomplishment (Maslach et al., 2001). Furthermore, emotional exhaustion has consistently been regarded as the most stable and informative indicator of individuals’ resource depletion under prolonged work stress (Roloff et al., 2022; Grayson and Alvarez, 2008). In educational settings, it is often reflected in emotional fatigue and negative attitudes toward teaching. Therefore, a growing body of research in the field of education has operationalized job burnout solely in terms of emotional exhaustion in empirical analyses (Kollerová et al., 2023; Schulze-Hagenest et al., 2023; Xu and Jia, 2022). From the perspective of the JD-R model, job burnout reflects the depletion of individuals’ emotional and psychological resources under sustained job demands. When teachers experience higher levels of job burnout, their occupational well-being tends to decrease (Hakanen et al., 2006). Therefore, the following hypothesis is proposed:

*H6*: Job burnout negatively influences occupational well-being.

**Figure 1:**
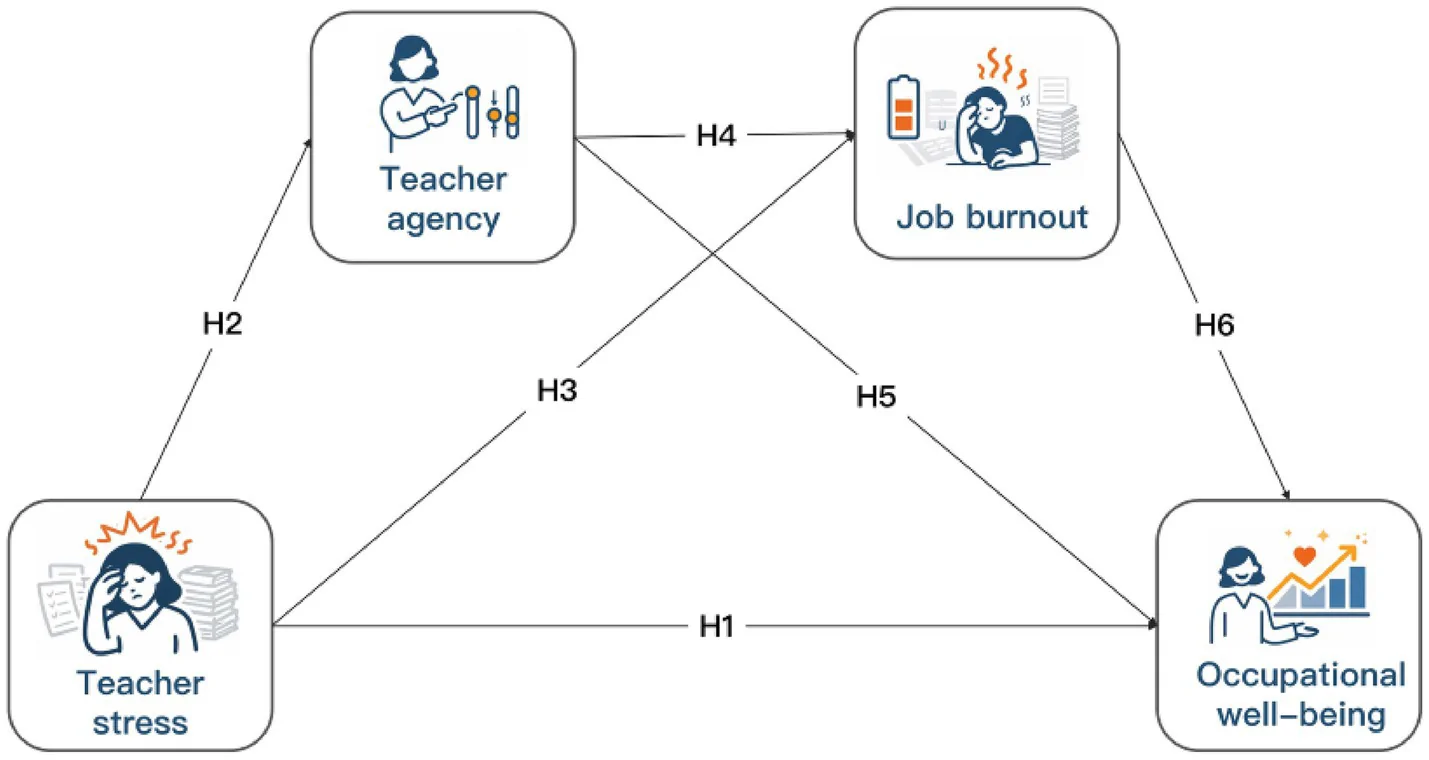
Conceptual model. Source: authors own work.

Based on the above theoretical arguments, the present study proposes that teacher stress influences occupational well-being through two interconnected processes involving proactive regulation and subsequent resource depletion. Specifically, teacher agency represents teachers’proactive behavioral responses to stressful job demands, whereas job burnout reflects the psychological consequences of prolonged resource depletion. Accordingly, the conceptual model presented in Figure 1 is proposed.

## 3 Methodology

### 3.1 Data collection and sample characteristics

This study was conducted in the Chinese educational context, where teacher stress has developed distinctive characteristics. Within China’s education system, teachers’ professional advancement is closely associated with academic qualifications, professional competence, and research achievements. Career progression is also shaped by performance-based evaluation systems, competition for limited professional resources, and standardized assessment requirements (Yang et al., 2019).

Data were collected through an online survey administered via the Wenjuanxing platform. Purposive sampling was employed to recruit participants, a method widely used in social science research, particularly for theory-testing studies (Sarstedt et al., 2018; Rather et al., 2024). Similar sampling strategies have been widely adopted in music education research, where participants (e.g., music teachers) are deliberately selected based on their professional experience and expertise (Creech, 2010). Given the absence of a comprehensive sampling frame for music teachers experiencing stress and occupational well-being, purposive sampling was considered both theoretically justified and methodologically appropriate.

The questionnaire targeted in-service primary and secondary school music teachers and was distributed to teachers with diverse educational backgrounds and demographic characteristics. To ensure the relevance of the sample to the research objectives, participants were required to meet the following criteria: (1) holding a music teaching position in a primary or secondary school; (2) perceiving their work environment as involving some degree of stress. All participants were formally employed teachers subject to annual performance evaluations, making them more likely to experience professional competition in teaching practice (Cheng et al., 2024).

To ensure the quality and validity of the online survey data, several screening procedures were implemented during data collection. At the beginning of the questionnaire, respondents were asked, “What type of school do you currently work in?” as an eligibility screening question. Only respondents who confirmed that they were in-service primary or secondary school music teachers were allowed to proceed with the subsequent sections of the questionnaire. Those who passed the screening were further invited to briefly describe their daily music teaching experiences and whether they perceived work-related stress. These open-ended responses were manually reviewed to identify suspicious cases, such as AI-generated responses, meaningless descriptions, or responses that were inconsistent with the participants’ reported teaching background, and questionnaires judged to be invalid were excluded from the final dataset. In addition, an instructional attention check item (e.g., “Please select “4 = Neutral” for this question.”) was embedded in the questionnaire to identify inattentive responding, and responses that failed this check were removed. These procedures helped exclude non-target participants and low-quality responses, thereby improving the relevance, validity, and overall quality of the final dataset.

To ensure the validity and usability of the measurement instrument, a pre-test and a pilot study were conducted prior to formal data collection. As the original scales were developed in English and applied in a Chinese context, a back-translation procedure was performed by two bilingual experts to ensure linguistic equivalence. Items with semantic inconsistencies were revised to improve clarity and accuracy. A pilot study with 50 participants was conducted to assess the measurement properties of the scales. Composite reliability (CR) ranged from 0.842 to 0.898, exceeding the recommended threshold of 0.70. Average variance extracted (AVE) ranged from 0.509 to 0.641, above the benchmark value of 0.50. These results indicate satisfactory reliability and convergent validity prior to the formal survey (Hair et al., 2019).

An *a priori* power analysis was conducted using G*Power 3.1. Following Cohen (2013) recommendation for a medium effect size, the effect size was set at *f*^2^ = 0.15, the significance level at *α* = 0.05, and the statistical power at 0.95. The calculation was based on the most complex endogenous construct in the structural model, which included three predictor variables (Hair et al., 2022). This procedure is also consistent with previous studies employing serial mediation models that have used G*Power to conduct an a priori power analysis (Jia et al., 2026). The analysis indicated that the minimum required sample size was 119. To further verify the adequacy of the sample size for the proposed serial mediation model, supplementary assessments were conducted using the inverse square root and gamma-exponential methods proposed by Kock and Hadaya (2018), together with a Monte Carlo power analysis. The inverse square root and gamma-exponential methods yielded recommended minimum sample sizes of 160 and 146, respectively, whereas the Monte Carlo power analysis for the serial indirect effect (a₁ × d × b₂) indicated that a minimum sample size of 107 was required to achieve a statistical power of 0.80. The final valid sample of 205 exceeded the minimum sample size recommended by all three methods, indicating that the present study had sufficient statistical power to test the proposed serial mediation model.

Demographically, female respondents accounted for 58.65% of the sample, while male respondents represented 41.35%. Participants were drawn from four types of schools: primary schools, middle schools, senior high schools, and secondary vocational schools, including both public and private institutions. Participants ranged in age from 22 to 45 years, with teaching experience ranging from 1 to 20 years. Most respondents held professional titles of Level 2 Teacher or Level 1 Teacher, positions that typically involve stable teaching responsibilities and regular performance evaluations.

### 3.2 Measures

All items were measured using a seven-point Likert scale (7-point Likert scale). The questionnaire measured four variables: teacher stress, teacher agency, job burnout, and occupational well-being. All measurement scales were adapted from existing validated scales and were appropriately modified according to the context of this study (see Appendix 2).

To improve the reliability and validity of the measurement instruments, this study followed existing methodological recommendations (Hinkin, 1998) and removed items with low factor loadings or insufficient reliability. Previous research has shown that, while maintaining internal consistency, retaining 3–5 high-quality items is generally sufficient to meet measurement requirements, and the marginal effect of continuously adding items on improving scale reliability is relatively limited (Hinkin, 1998).

Teacher Stress was measured using the scale developed by Chen et al. (2022). The original scale contained 9 items, and 8 items were retained in this study. A sample item is TS01: “I’m feeling under a lot of pressure because I have too much teaching work (music activities, music competitions).” Item TS07 from the original scale was removed.

Teacher Agency was measured using the scale developed by Pietarinen et al. (2013). The original scale contained 7 items and included two dimensions: self-regulation and co-regulation. In this study, four items were removed based on the measurement assessment, and a final set of 3 items was retained for analysis. A sample item is TA01: “I try out new ideas when I am doing a routine task.”

Job Burnout was measured using the Emotional Exhaustion dimension of the Maslach Burnout Inventory (MBI) developed by Maslach and Jackson (1981). Emotional exhaustion is widely regarded as the core dimension of job burnout, which can reflect the continuous depletion of individuals’ psychological resources under long-term work stress and is commonly used as an operational measure of job burnout in empirical research (Wang, 2020). The original Emotional Exhaustion dimension contained 9 items, and 5 items were retained in this study for analysis. A sample item is JB01: “I feel quite exhausted from teaching.” Items 3, 4, 5, and 7 from the original scale were removed.

Occupational Well-being was measured using the Teacher Subjective Well-Being Questionnaire (TSWQ) developed by Renshaw et al. (2015). The original questionnaire contained 12 items covering three dimensions: School Connectedness, Joy of Teaching, and Teaching Efficacy. In this study, 4 items were removed, and a final set of 8 items was retained for analysis. A sample item is OWB01: “I really enjoy working with students.”

To examine the quality of the measurement model, this study systematically tested the reliability and validity of each construct based on PLS-SEM measurement model evaluation criteria. First, indicator reliability was assessed using item outer loadings, which should be greater than 0.708. Second, composite reliability should be above 0.70, and AVE should be greater than 0.50 (Hair et al., 2019). Finally, discriminant validity assessed by HTMT values below 0.90 indicates good discriminant validity between latent variables (Henseler et al., 2015).

### 3.3 Data analysis

This study employed variance-based Partial Least Squares Structural Equation Modeling (PLS-SEM) for data analysis. PLS-SEM is capable of simultaneously estimating both the measurement model and the structural model and is suitable for handling theoretical models involving multiple latent variables and complex path relationships (Hair et al., 2019). Therefore, PLS-SEM is well aligned with the model structure and research objectives of this study. The overall analytical procedure consisted of common method bias assessment, measurement model assessment, structural model assessment, and nonlinear effect analysis.

Given that this study employed a cross-sectional questionnaire survey design, which may introduce common method bias (CMB), the first step of the data analysis was to assess the potential presence of common method bias. Next, the measurement model was assessed within the PLS-SEM framework. Composite Reliability (CR) and Average Variance Extracted (AVE) were used to evaluate internal consistency reliability and convergent validity, respectively, while the Heterotrait–Monotrait Ratio (HTMT) was employed to assess discriminant validity among the constructs (Hair et al., 2019). Subsequently, the structural model was assessed. First, multicollinearity among the predictor constructs was examined to ensure the reliability of the structural model estimation. Then, a bootstrapping procedure with 5,000 resamples was performed to estimate the significance of the path coefficients and to further test the direct and mediating effects (Hair et al., 2021). Finally, to further examine the potential nonlinear relationship between teacher stress and occupational well-being, a quadratic term of teacher stress was incorporated into the original structural model for exploratory analysis to test the potential nonlinear effect.

## 4 Results

### 4.1 Common method bias

This study employed a cross-sectional design, which may introduce common method bias. Following Podsakoff et al. (2012), both procedural and statistical remedies were applied. For procedural remedies, expert input was incorporated in the questionnaire design, and respondent anonymity and data confidentiality were ensured to reduce potential bias. A pilot test was conducted, and measurement items were refined to improve clarity and readability (Podsakoff et al., 2003).

Regarding statistical testing, a full multicollinearity test was first conducted (Kock, 2017). The results indicated that the values of the variance inflation factor ranged from 1.191 to 1.511, all of which were below the recommended threshold of 3.3 (Kock, 2017). Furthermore, within the framework of a partial least squares structural equation model, this study employed latent variable correlation methods to test for common method bias (Bagozzi and Phillips, 1991). The results indicated that the correlation coefficients between all latent variables were below the threshold of 0.90, suggesting that there were no serious issues of common method variance arising from high inter-construct correlations, and that the risk of common method bias was low.

### 4.2 Measurement model assessment

The composite reliability (CR) values of all constructs reached satisfactory levels, ranging from 0.794 to 0.911, all exceeding the recommended threshold of 0.70. The average variance extracted (AVE) values ranged from 0.553 to 0.672, with all constructs exceeding the benchmark value of 0.50 (see Table 1). Therefore, the results confirm that the measurement model demonstrates adequate convergent validity (Hair et al., 2019). Discriminant validity was assessed using the heterotrait–monotrait ratio (HTMT) approach (see Table 2). All HTMT values are below the recommended threshold of 0.90, indicating that satisfactory discriminant validity is established among the constructs.

**Table 2:** Discriminant validity heterotrait-monotrait ratio.

	Teacher stress	Job burnout	Occupational well-being	Teacher agency
Teacher stress				
Job burnout	0.310			
Occupational well-being	0.577	0.577		
Teacher agency	0.694	0.528	0.746	

Source: authors own work.

### 4.3 Structural model assessment

The assessment of the structural model began with an examination of potential collinearity issues. The results indicate that the variance inflation factor (VIF) values for all constructs ranged from 1.059 to 2.156, all of which are below the critical threshold of 3.3, suggesting that no significant multicollinearity issues exist in the model (Hair et al., 2021).

**Figure 2:**
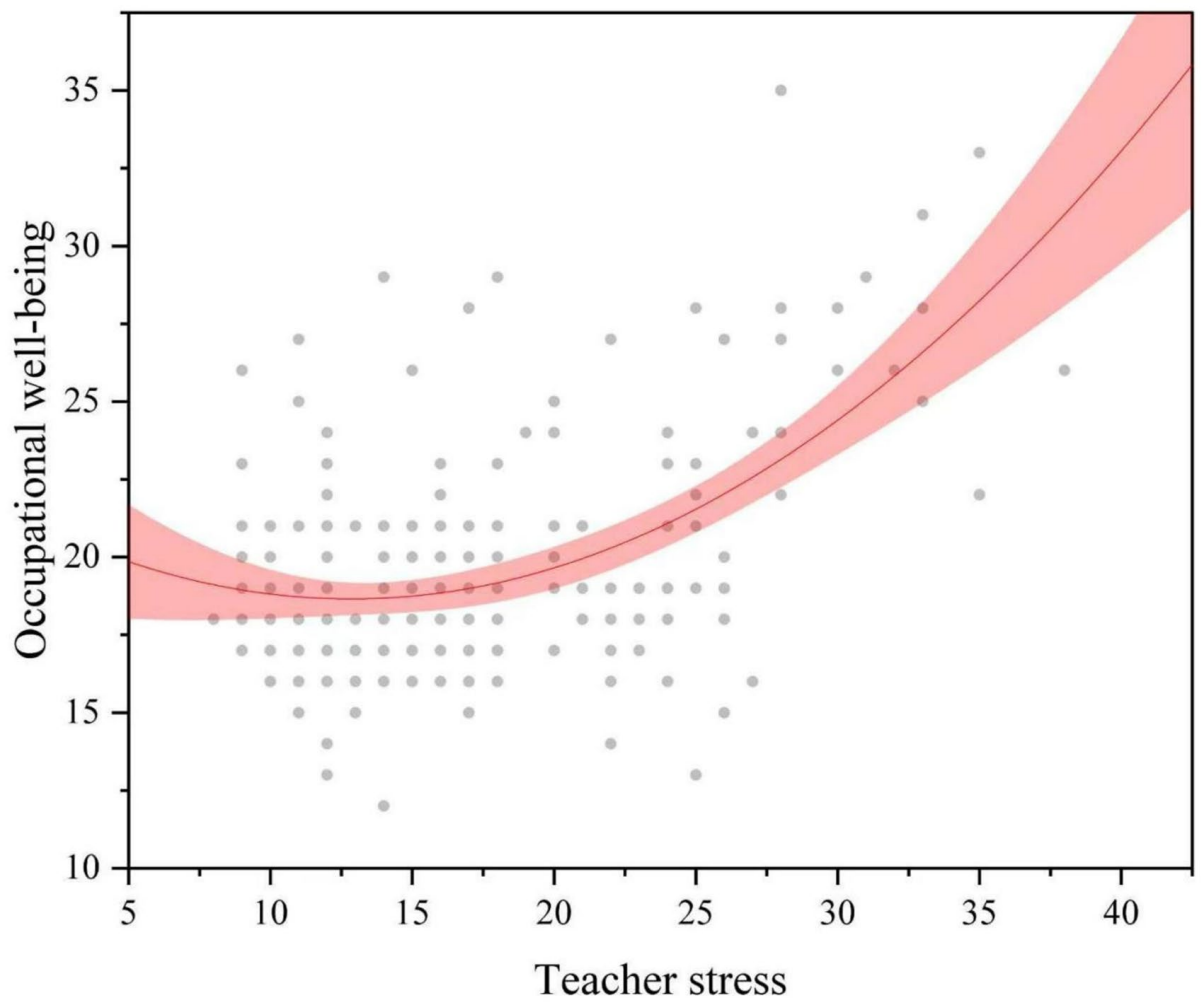
Quadratic relationship between teacher stress and occupational well-being.

Structural model evaluation was conducted using the bootstrapping procedure with 5,000 resamples (see Table 3). Regarding direct effects, four out of the six proposed hypotheses were supported (Figure 2). Teacher stress had a significant positive effect on occupational well-being (*β* = 0.259, *p* < 0.01); however, as the hypothesized relationship was negative, H1 was not supported. Teacher stress also exerted a significant positive influence on teacher agency (*β* = 0.582, *p* < 0.01), supporting H2. In contrast, teacher stress did not have a significant effect on job burnout (*β* = −0.064, *p* > 0.05), and thus H3 was not supported. Teacher agency had a significant negative effect on job burnout (*β* = −0.360, *p* < 0.01), supporting H4. Additionally, teacher agency positively influenced occupational well-being (*β* = 0.275, *p* < 0.01), supporting H5. Job burnout had a significant negative effect on occupational well-being (*β* = −0.324, *p* < 0.01), supporting H6.

**Table 3:** Hypothesis testing.

Hypothesis	Relationship	Std.*β*	*t*-value	Effect size	Decision
H1	TS- > OWB	0.259	3.532**	0.080	Unsupported
H2	TS- > TA	0.582	11.694**	0.511	Supported
H3	TS- > JB	−0.064	0.752 ^n.s^.	0.003	Unsupported
H4	TA- > JB	−0.36	4.060**	0.102	Supported
H5	TA- > OWB	0.275	3.445**	0.082	Supported
H6	JB- > OWB	−0.324	4.950**	0.159	Supported

*p < 0.05, **p < 0.01, ^n.s.^, not significant.

TS, Teacher stress; TA, Teacher agency; JB, Job burnout; OWB, Occupational well-being.

Source: authors own work.

**Figure 3:**
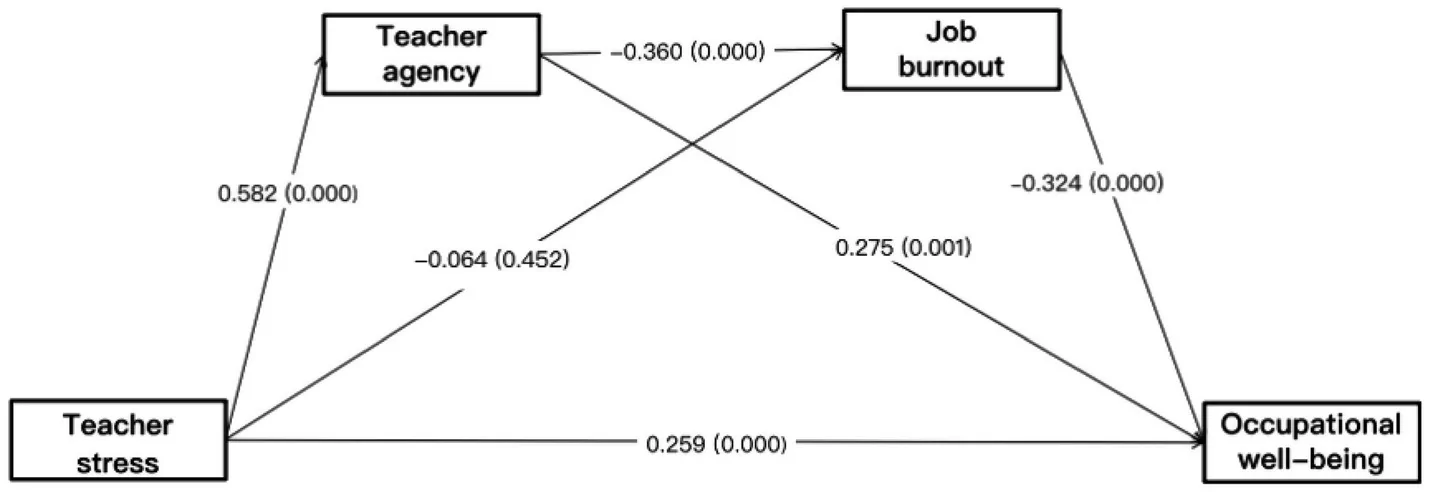
Main effects.

Regarding indirect effects (see Table 4), the indirect effect of teacher agency on occupational well-being through job burnout was significant (*β* = 0.117, *p* < 0.01). In contrast, the mediating effect of job burnout in the relationship between teacher stress and occupational well-being was not significant (*β* = 0.021, *p* > 0.05). Teacher stress showed a significant indirect effect on job burnout through teacher agency (*β* = −0.210, *p* < 0.01). In addition, the indirect effect of teacher stress on occupational well-being through teacher agency was significant (*β* = 0.160, *p* < 0.01). The serial mediation effect of teacher stress on occupational well-being via teacher agency and job burnout was also significant (*β* = 0.068, *p* < 0.01). These results indicate that teacher agency plays a central mediating role, while job burnout functions primarily within the serial mediation pathway Figure 3.

**Table 4:** Mediation and conditional indirect effects.

Relationship	Std.*β*	*t*-value	Effect size	95% bias-corrected		Percentile 95%	
				Lower limit	Upper limit	Lower limit	Upper limit
TA- > JB- > OWB	0.117	3.009^**^	0.117	0.058	0.188	0.054	0.181
TS- > JB- > OWB	0.021	0.735^n.s.^	0.021	−0.023	0.066	−0.024	0.065
TS- > TA- > JB	−0.210	3.885^**^	−0.210	−0.299	−0.121	−0.302	−0.124
TS- > TA- > OWB	0.160	3.255^**^	0.160	0.064	0.224	0.060	0.220
TS- > TA- > JB- > OWB	0.068	2.935^**^	0.068	0.033	0.110	0.031	0.107

^*^p < 0.05, ^**^p < 0.01^, n.s.^, not significant.

TS, Teacher stress; TA, Teacher agency; JB, Job burnout; OWB, Occupational well-being.

Source: authors own work.

To further examine the potential nonlinear impact of teacher stress on occupational well-being, this study conducted a post-hoc exploratory analysis based on the original model, incorporating a quadratic term of teacher stress into the model for testing (see Table 5). The results show that the quadratic term of teacher stress has a significant positive impact on occupational well-being (*β* = 0.112, *p* < 0.05), with a 95% confidence interval of [0.036, 0.205], and does not include zero, indicating that the effect is statistically significant. These findings suggest that the association between teacher stress and occupational well-being cannot be fully explained by a simple linear model and may instead follow a nonlinear pattern. As illustrated in Figure 2, occupational well-being showed a slight decline as teacher stress increased from low to moderate levels, reaching its minimum at approximately moderate stress levels. Beyond this point, occupational well-being increased progressively with higher levels of teacher stress. Overall, the fitted quadratic curve suggests a modest U-shaped relationship between teacher stress and occupational well-being.

**Table 5:** Results for quadratic effect exploration.

Relationship	Std. *β*	Standard deviation	T value	*p* values	[95% conf. interval]
QE (TS) - > OWB	0.112	0.052	2.136	0.016	[0.036,0.205]

QE, quadratic effect; TS, Teacher stress; OWB, Occupational well-being.

Source: authors own work.

## 5 Discussion

Based on an empirical analysis of 205 in-service primary and secondary school music teachers in China, this study found that teacher stress is positively associated with occupational well-being. This differs from previous studies that primarily view stress as a negative factor (Chen, 2016; Shao et al., 2025). This suggests that teacher stress does not show a single negative association with occupational well-being, but rather indirectly through individuals’ cognitive appraisal and behavioral responses. Based on the challenge–hindrance stress framework (LePine et al., 2004), stress itself does not directly determine individuals’ occupational well-being; its effect depends on how individuals understand and cope with stressful situations. When teachers perceive stress as a manageable or meaningful challenge, it may be associated with higher levels of work engagement and goal-oriented motivation (Chan and Hui, 1995), may be associated with higher work-related experience. Therefore, the positive relationship observed in this study is more likely to reflect an indirect process in which stress acts as a situational stimulus through individuals’ adaptive responses, rather than reflecting a direct association.

Furthermore, the quadratic term of teacher stress reached statistical significance, suggesting that the relationship between teacher stress and occupational well-being may not be fully explained by a simple linear assumption and may involve a potential nonlinear pattern. This finding echoes the broader proposition of the Yerkes–Dodson law that the relationship between stress-related arousal and individual outcomes may be nonlinear rather than purely linear (Yerkes and Dodson, 1908; Teigen, 1994). However, because the Yerkes–Dodson law primarily concerns task performance, whereas the present study focuses on occupational well-being as a subjective psychological outcome, the present finding should not be interpreted as a direct extension of that framework or as evidence that higher levels of teacher stress necessarily enhance intrinsic motivation. Rather, the result suggests that teacher stress, as a global construct, may involve both motivational and resource-related processes. From the perspective of SDT, autonomous motivation is associated with more self-regulated and autonomous forms of behavior (Baard et al., 2004), which may help explain why teacher stress was positively associated with teacher agency in this study. At the same time, the JD-R model suggests that sustained job demands may consume individuals’ psychological resources and increase the risk of burnout (Bakker and Demerouti, 2017), although the weak direct association between teacher stress and job burnout in the present study indicates that stress alone may not inevitably translate into emotional exhaustion.

One possible explanation is that the observed nonlinear relationship between teacher stress and occupational well-being may reflect the joint influence of different psychological processes. When stress remains relatively manageable, teachers may maintain their occupational well-being through autonomous regulation and proactive coping, which is consistent with the self-regulatory processes emphasized by SDT. In contrast, when stress accumulates over time and exceeds the resources available to the individual, the resource depletion process proposed by the JD-R model may become increasingly dominant. Because the nonlinear analysis in the present study was conducted as a *post hoc* exploratory analysis, this interpretation should be considered tentative and requires further empirical verification in future research.

Teacher agency plays a significant mediating role between teacher stress and job well-being. This finding is consistent with existing research showing that a positive relationship exists between teacher agency and job well-being (Yüce et al., 2024). Building on this, the present study further reveals the mediating mechanism of teacher agency in the relationship between teacher stress and job well-being. Specifically, under a certain level of work stress, teachers do not passively bear stress but tend to actively adjust their teaching behaviors and increase their professional engagement to cope with external demands, thereby being associated with higher job well-being and sense of accomplishment. From the perspective of SDT, individuals maintain intrinsic motivation by enhancing autonomy and competence when facing external stress, a process that helps improve their work-related well-being. Therefore, this study shows that stress does not necessarily reduce job well-being, but under certain conditions, it can be transformed into a positive outcome by stimulating individual initiative.

This study further finds that teacher stress is associated with occupational well-being through a chain associative pathway involving teacher agency and job burnout. Specifically, teacher stress is not directly associated with job burnout; rather, its subsequent impact is shaped through the mediating role of teacher agency. This finding is consistent with the perspective of Bakker and Demerouti (2007), which suggests that teacher stress does not necessarily has a direct link to job burnout, and that individual regulatory processes play an important role in shaping its effects. As an important psychological resource, teacher agency is associated with individuals’ ability to maintain a high level of psychological resilience when facing work-related stress and to actively regulate their behaviors in response to external demands. Through this process, teacher agency can, to some extent, weaken or buffer the association between stress and job burnout. At the same time, higher levels of teacher agency reflect stronger self-regulation capacity under high job demands, thereby being associated with lower levels of emotional exhaustion (Wang et al., 2024). Therefore, stress does not inevitably develop into negative outcomes. Instead, under the influence of teacher agency, its effects can be redirected and, to a certain extent, less likely to be associated with job burnout, thereby helping to maintain and enhance occupational well-being.

Contrary to H3, the present study found that the direct association between teacher stress and job burnout was not significant. This finding suggests that the relationship between teacher stress and job burnout may be more complex than that predicted by the traditional JD-R model. Although the JD-R model proposes that sustained job demands ultimately lead to job burnout through the process of resource depletion, the present study, by integrating SDT, suggests that this process may not occur immediately, but may first involve teachers’ proactive regulation. Based on the integrated theoretical framework of the JD-R model and SDT adopted in the present study, when teachers face work-related stress, they tend to first respond to job demands by adjusting their teaching practices, increasing their professional engagement, or actively utilizing available resources, thereby demonstrating higher levels of teacher agency. When teachers possess stronger coping capacity or have access to sufficient resource support, work-related stress may be alleviated through the process of proactive regulation rather than further developing into emotional exhaustion and job burnout (Rinne et al., 2022). Only when work-related stress persists and effective coping strategies or resource support are lacking is stress more likely to gradually lead to resource depletion and eventually develop into job burnout (Shaw, 2016). Therefore, the occurrence of job burnout cannot be directly explained by stress itself, but is jointly shaped by individuals’ coping processes and resource conditions. This may also explain why the association between teacher stress and job burnout did not reach statistical significance in the present study.

This finding further suggests that, when explaining the mechanisms through which teacher stress operates, it may not be sufficient to understand the effects of stress solely from the perspective of resource depletion. It is also necessary to consider teachers’ proactive coping and behavioral regulation processes under stressful conditions. Therefore, the present study supports the necessity of integrating SDT and the JD-R model to understand the mechanisms through which teacher stress operates, and further suggests that future research should pay greater attention to the boundary conditions underlying the relationship between teacher stress and job burnout.

### 5.1 Theoretical implication

This study extends the theoretical understanding of the relationship between teacher stress and occupational well-being. The findings show that teacher stress is not only significantly associated with occupational well-being, but also exhibits a clear non-linear pattern. This result indicates that the relationship between teacher stress and occupational well-being does not follow a simple “more stress, lower well-being” pattern, but instead reflects a dynamic form of influence, thereby challenging prior research that predominantly conceptualizes teacher stress from a single negative perspective. It further suggests that the relationship between teacher stress and outcomes is context-dependent, with its associations varying across different levels of stress, thus enriching the understanding of stress-related mechanisms.

In addition, this study develops a serial mediation model by introducing teacher agency and job burnout as mediating variables, thereby examining the process through which teacher stress is associated with occupational well-being. Compared with existing research that primarily focuses on the direct relationship between stress and outcome variables, this study shows that the associations between teacher stress and outcomes are linked through a series of mediating processes, extending the understanding of how stress operates. Although prior studies have examined the relationship between stress and resource depletion, limited attention has been paid to individuals’ proactive regulation under stressful conditions. By incorporating teacher agency as a key variable, this study highlights its linking role in the stress process and suggests that teacher stress not only operates through a resource depletion pathway, but also relates to differentiated outcomes through an agency-based mechanism.

### 5.2 Practical implication

This study provides important practical implications for the professional development of music teachers and the sustainability development of the education system. The findings suggest that simply reducing competition or lowering work stress may not be sufficient to improve teachers’ occupational well-being. Instead, greater attention should be paid to the structural management of stress. Given the non-linear relationship between teacher stress and occupational well-being, educational administrators are encouraged to avoid avoid adopting a one-size-fits-all stress reduction approach and to consider the distribution and intensity of workload more carefully. For example, excessive performance evaluation indicators may be streamlined, the balance between teaching and administrative tasks may be adjusted, and phased work goals can be established to maintain an appropriate level of challenge. Such measures may support sustain teachers’ engagement and intrinsic motivation and relate to the long-term sustainability of teachers’ occupational well-being, rather than suppressing stress indiscriminately.

The findings indicate the critical role of teacher agency in educational practice. Rather than relying solely on external support, schools should actively foster teachers’ capacity for proactive regulation. This may be supported by expanding teachers’ autonomy in curriculum design and instructional planning, establishing institutional channels for teacher participation in decision-making, and offering reflective professional development opportunities that help teachers better interpret and manage stress. Moreover, differentiated support strategies may be considered in response to varying stress levels. For instance, collaborative teaching support and psychological resources can be provided under high-stress conditions, while goal-oriented guidance may be more appropriate when stress levels are moderate. Such approaches may be associated with stress being reframed as a source of professional growth and support the sustainable maintenance of teachers’ occupational well-being over time.

### 5.3 Limitation and future research directions

This study has several limitations that provide directions for future research. First, this study adopted a cross-sectional research design, which limits the inference of causal relationships among the variables. Although the proposed relationships were developed based on theoretical foundations, the causal direction cannot be rigorously verified. In terms of the measurement approach, data collected at a single time point may limit the ability to capture dynamic changes in psychological states. Constructs such as teacher stress, teacher agency, and occupational well-being may exhibit temporal fluctuations and context dependence in real-world settings, whereas a single measurement cannot reflect their developmental trajectories and cumulative effects over time. Future research could adopt a longitudinal design to further examine these relationships.

Second, this study used data collected through an online questionnaire. On the one hand, the sample size was relatively limited, which may affect the robustness of the statistical results. On the other hand, the online data collection method may introduce sampling bias due to its limited coverage and the effect of voluntary participation, thereby limiting the representativeness of the sample. Future research could incorporate data from multiple sources, combine online and offline multi-channel sampling methods, and further increase the sample size to improve the reliability and robustness of the research findings.

Third, teacher agency was measured using three retained items from the original seven-item scale developed by Pietarinen et al. (2013). Although these items demonstrated acceptable psychometric properties, a three-item measure may not fully capture the conceptual complexity of teacher agency. Future research could employ more comprehensive teacher agency measures to further validate the robustness of the present findings.

Although this study reveals the mediating role of teacher agency in the relationship between teacher stress and occupational well-being, the formation of teacher agency is not systematically examined. This limits a more in-depth understanding of the underlying mechanisms through which stress operates. Future research could investigate antecedents of teacher agency, such as organizational support, leadership style, or institutional environment, to extend the theoretical framework.

## Data Availability

The raw data supporting the conclusions of this article will be made available by the authors, without undue reservation.
