# Supplementary material for: Beyond the negative view of music teacher stress: how stress enhances occupational well-being through teacher agency
Source: Front Psychol. 2026 Sep 2;17:1865707. doi: 10.3389/fpsyg.2026.1865707 (PMC13579791; doi:10.3389/fpsyg.2026.1865707)
Supplement: Supplementary file 1 [file Data_Sheet_1.PDF]

Appendix 1. Relative research on occupational wellbeing

Articles	Independent variables / mediators/ moderators	Dependent variables	Theory/model applied	Research type	Research methodology and sampling method	Research origin	Research perspective	Key findings	Distinctive features of this study
MacIntyre et al. (2019)	•Independent variable Personality traits Stressors	Teacher wellbeing	Teacher wellbeing	Quantitative	Online survey Non-probability sampling	International sample (17 nationalities, 22 countries)	Individual differences	Wellbeing was negatively related to chronic stressors and stressful life events, while agreeableness and emotional stability were the strongest positive predictors. Personality showed limited links to stress exposure overall. Interpersonal relationships, especially teacher student relationships, were the key factor in teacher satisfaction, and the PERMA dimensions were highly interrelated rather than independent.	This study addresses a key limitation in research on teachers' occupational well-being: although a substantial body of literature has examined relevant antecedents, findings regarding the effects of stress remain inconsistent. Existing studies have explored various influencing factors such as mental health, transformational leadership, and school climate; however, stress is often conceptualized in a simplistic manner as a purely negative factor, and

Shao et al. (2025)	•Independent variable Work stress •Mediator Affective rumination Work engagement	Well-being	Job demands-resources model	Quantitative	Paper questionnaire Cluster sampling	China	Occupational stress and resource	Work stress has a significant direct negative impact on happiness, and it also influences happiness indirectly through emotional rumination and work engagement. Specifically, emotional rumination and work engagement each serve as mediators, and together they form a chain mediating pathway linking work stress to happiness.	the analyses largely remain at the level of variable associations. In contrast, the present study examines the underlying mechanisms of stress and highlights individuals' active regulatory roles under stressful conditions. This suggests that the relationship between teacher stress and occupational well-being is not merely a simple linear negative association, and its mechanisms warrant further investigation.
Wang et al. (2024)	•Independent variable Self-efficacy •Mediator Teaching satisfaction Resilience	Teachers' well-being	Subjective well-being theory	Mixed	Online survey Semi-structured interviews	China	Positive psychology resources	Self-efficacy significantly predicted teaching satisfaction, resilience, and well-being, while both teaching satisfaction and resilience were significant predictors of well-being. Moreover, teaching satisfaction and resilience each mediated the relationship between self-efficacy and	Building on this research gap, the present study overcomes the limitations of relying on a single theoretical framework by integrating Self-Determination Theory (SDT) and the Job Demands–Resources (JD-R) model. Prior research has

								well-being.	primarily adopted a resource-depletion perspective to explain the effects of stress, whereas this study adopts a dual lens of resources and motivation, emphasizing that stress may simultaneously entail both
Rinne et al. (2022)	•Independent variable Personal background Work-related Overall occupational well-being	Individual aspect of occupational well-being	Content model for the promotion of school Community staff's occupational well-being	Quantitative	Online survey Cross-sectional survey design	Finland	Individual-level （physical and mental workability)	Resources for managing mental workload and workplace support were both rated as quite poor, while resources for physical workload received the highest ratings and occupational health care services were assessed at a moderate level.	depletion and activation effects, thereby highlighting its multidimensional nature and potential positive implications for teachers' occupational well-being. From a research perspective, this study further addresses the lack of attention to behavioral processes in prior literature by incorporating
Zhen and Liu (2023)	•Independent variable Teacher professional identity •Mediator Psychological	Teacher occupational happiness	Occupational identity theory Theory of psychological capital	Quantitative	Online survey Random sampling	China	Psychological capital and professional identity	Occupational identity has a significant positive impact on both psychological capital and occupational happiness, while psychological capital also positively predicts	teacher agency as a key analytical variable within the conceptual framework. Whereas existing studies have mainly focused on passive psychological

	capital							occupational happiness and fully mediates the relationship between occupational identity and occupational happiness.	responses to stress, this study examines teachers' active self-regulatory processes and reveals the critical mediating role of teacher agency in the relationship between stress and occupational well-being.
Han et al. (2020)	•Independent variable<br>Challenge job demands<br>Job resources<br>•Moderators<br>Teacher efficacy	Negative indicator of well-being<br>Work engagement	Job demands–resources	Quantitative	Online survey<br>Stratified sampling<br>Cross-sectional survey design	China	A job demands–resources perspective focusing on the mediating role of personal resources in teachers' occupational well-being	Challenge job demands were positively related to emotional exhaustion and occupational burnout and negatively related to work engagement, whereas job resources were positively associated with work engagement and negatively related to emotional exhaustion. In addition, teacher efficacy was positively related to both challenge job demands and work engagement.	Through the integration of multiple theoretical perspectives, this study extends the explanatory pathways of teacher stress mechanisms and emphasizes the dynamic processes through which stress operates. This theoretical shift contributes to a more nuanced understanding of the complex and context-dependent relationship between teacher stress and occupational well-being.

Sun et al. (2025)	•Independent variable Presidents' transformational leadership •Mediator Job crafting Teaching efficacy	Faculty occupational well-being	Job demands–resources Transformational leadership theory	Quantitative	Online survey Random sampling Cross-sectional survey design	China	Transformational leadership perspective with a mediation mechanism approach	Presidents' transformational leadership significantly and positively predicted faculty occupational well-being, with both job crafting and teaching efficacy serving as partial mediators in this relationship.
Si, J. (2024)	Not applicable	Teachers' professional well-being	Well-being theory Managerialism theory	Qualitative	Policy document analysis Open-ended questionnaires Semi-structured Interviews Convenience sampling	China	Managerial culture	Teachers in this study reported that managerialism profoundly undermines their professional well-being. The abandonment of traditional job security and the introduction of tenure track have created pervasive job insecurity, with language teachers facing layoffs when their disciplines are deemed low in market value.

Kiltz et al. (2020)	Not applicable	Student well and Teacher well-being	Self-determination theory Resilience theory	Qualitative	Semi-structured Interviews Purposive sampling	Germany and the Netherlands	Student–teacher interaction perspective	Student and teacher well-being are strongly interconnected. The student–teacher relationship is the central mechanism through which well-being flows between the two groups. Well-being includes physical, mental, social, and meaningful engagement, not just the absence of stress.
Kim and Asbury (2021)	Not applicable	Teachers' mental health and well-being	Job demands–resources model	Qualitative	Semi-structured Interviews Purposive sampling	England	A JD-R–based longitudinal qualitative perspective on teachers' mental health and well-being	Teachers' mental health and well-being generally declined throughout the pandemic, with a non-linear "rollercoaster" pattern. Three job resources were identified as positively contributing to mental health and well-being: social support, work autonomy, coping strategies.

Hascher and Waber (2021)	Not applicable	Teacher well-being	Psychology of well-being positive psychology Psychology of work and organisation	Systematic review	Data collection	Studies from over 40 countries	Systematic review perspective on teacher well-being	Teacher well-being research spans five distinct theoretical fields, with over 90 different instruments used, complicating comparison and synthesis.
Han and Gao (2023)	•Independent variable Teaching support •Mediator Teacher self-efficacy	Teacher occupational well-being	Interactionist model of teacher well-being	Quantitative	Online survey Convenience sampling	China	Teaching support (external resources)	Teaching support (especially resources and autonomy) enhances teacher self-efficacy and engagement, while self-efficacy partially mediates the relationship between support and teacher well-being. Information and communication technology enhanced teaching does not lead to significant emotional exhaustion.

Zhang et al. (2024)	Not applicable	Teacher's well-being and professional behaviors	Not applicable	Quantitative	Semi-structured Interviews	China	Institutional and policy perspective (policy driven, performance-oriented)	Performance-based pay and tenure-track systems have significantly reshaped teachers' professional behaviors. The academic environment has become highly competitive and unhealthy, leading to increased occupational stress and a decline in teachers' occupational well-being.

Source: Source: Authors own work.

Appendix 2. Measurement items

Variable	Scale	Source of Scale	Final items
Teacher stress	TS1: I felt stressed when encountering issues with my students' families.<br>TS2: I felt stressed for not having support from the administrators at my school.<br>TS3: I felt stressed for not having support from colleagues at my school.<br>TS4: I felt stressed for not having support from my family and friends.<br>TS5: I felt stressed for having to manage student behaviors.<br>TS6: I felt stressed for having too much teaching work to do.<br>TS7: I felt stressed for not having enough time to complete my teaching work (e.g., preparing, teaching the curricular content).<br>TS8: I felt stressed for not being able to meet the diverse learning needs of my students.<br>TS9: I felt stressed about not doing a good job with my teaching.	Chen et al. （2022）	TS01: I felt stressed when encountering issues with my students' families.<br>TS02: I felt stressed for not having support from the administrators at my school.<br>TS03: I felt stressed for not having support from colleagues at my school.<br>TS04: I felt stressed for not having support from my family and friends.<br>TS05: I felt stressed for having to manage student behaviors.<br>TS06: I'm feeling under a lot of pressure because I have too much teaching work (music activities, music competitions).<br>TS07: I felt stressed for not being able to meet the diverse learning needs of my students.<br>TS08: I felt stressed about not doing a good job with my teaching.

Teacher agency	TA1: I'm able to control my work pace in the busy school work schedule.<br>TA2: I can set limits to my work assignments.<br>TA3: I know when it's time for me to adjust my work pace.<br>TA4: It's possible to learn to adjust the way you manage your work strain.<br>TA5: I'm able to support the colleagues who feel strain in their work.<br>TA6: I'm asking my colleagues for support when facing exhausting work situations.<br>TA7: I'm getting better and better in recognising the situations in which I have succeeded as a teacher.	Pietarinen et al. (2013)	TA01: I'm able to control my work pace in the busy school work schedule.<br>TA02: I can set limits to my work assignments.<br>TA03: I'm able to support the colleagues who feel strain in their work.
Job burnout	JB1: I feel emotionally drained from my work.<br>JB2: I feel used up at the end of the workday.<br>JB3: I feel fatigued when I get up in the morning to face another day on the job and have a strain.<br>JB4: Working with people all day is really for me.<br>JB5: I feel burned out from my work.<br>JB6: I feel frustrated by my job.<br>JB7: I feel I'm working too hard on my job.<br>JB8: Working with people directly puts too much stress on me.<br>JB9: I feel like I'm at the end of my rope.	Maslach and Jackson (1981)	JB01: I feel emotionally drained from my work.<br>JB02: I feel used up at the end of the workday.<br>JB03: I feel frustrated by my job.<br>JB4: Working with people directly puts too much stress on me.<br>JB5: I feel like I'm at the end of my rope.

Occupational well being	OWB1: I feel like I belong at this school.<br>OWB2: I can really be myself at this school.<br>OWB3: I feel like people at this school care about me.<br>OWB4: I am treated with respect at this school.<br>OWB5: I get excited about teaching and learning.<br>OWB6: I am very interested in the things we are doing at this school.<br>OWB7: I really enjoy working with students.<br>OWB8: I feel happy when I am working at this school.<br>OWB9: I am a successful teacher.<br>OWB10: I am good at helping students learn new things.<br>OWB11: I have accomplished a lot as a teacher.<br>OWB12: I feel like my teaching is effective and helpful.	Renshaw et al. （2015）	OWB1: I get excited about teaching and learning.<br>OWB2: I am very interested in the things we are doing at this school.<br>OWB3: I really enjoy working with students.<br>OWB4: I feel happy when I am working at this school.<br>OWB5: I am a successful teacher.<br>OWB6: I am good at helping students learn new things.<br>OWB7: I have accomplished a lot as a teacher.<br>OWB8: I feel like my teaching is effective and helpful.

Source: Source: Authors own work.
